# *Irvingia gabonensis *fat: nutritional properties and effect of increasing amounts on the growth and lipid metabolism of young rats *wistar *sp

**DOI:** 10.1186/1476-511X-10-43

**Published:** 2011-03-04

**Authors:** Thierry Joël Nangue, Hilaire Macaire Womeni, Felicite Tchouanguep Mbiapo, Jacques Fanni, Linder Michel

**Affiliations:** 1 Laboratory of Food Sciences Nutrition and Medicinal Plant, Department of Biochemistry, Faculty of Science, University of Dschang, P.O. BOX: 67 Cameroon; 2 Laboratory of Biomolecular Engineering (lIbio), National School of Agronomy and Food Industries ENSAIA - INPL, 2 Avenue de la Forêt de Haye BP 172 F-54505 Vandoeuvre-lès-Nancy, France

## Abstract

**Background:**

Dietary saturated fatty acids (SFAs) are generally considered to increase plasma cholesterol. It has also been claimed that they increase cardio-vascular disease, although the claim that some of SFAs can increase HDL-cholesterol is poorly documented. *Irvingia gabonensis *kernels after being dried and crushed they are generally used to prepare a sticky and aromatic soup very much consumed in Cameroun and West Africa countries. This study was therefore aimed at evaluating the effects of dika nut fat on the growing and lipids metabolism of young rats.

**Method:**

For The nutritional evaluation related to the performances of growth and the analysis of increasing amounts of dika nut fat (0; 5.1; 7.34 and 13.48%) in young rats of *wistar sp*. The animals were taken individually out of metabolic cage for each ration 5 repetitions per sex (males and females) were carried out.

**Results:**

The results obtained during the 3 weeks of treatment shows that the performances of consumption were positive. A highly significant increase (P < 0,01) of serum cholesterol and triglycerides in the high dose fat groups (13,48%) of dika fat were observed compared to control groups. However, this rise of cholesterol is due to that of HDL-cholesterol without any change in the quantity of LDL-Receptor. In parallel, the weight of the vital organ did not vary much compared to control, except for males where we observed a significantly reduction (P < 0,01) in the weight of the liver for the three diet tests.

**Conclusion:**

This study shows that the increasing amount of dika nut fat alter significantly cholesterol and triglyceride at high dose diet, but also increase HDL-cholesterol.

## 1 - Background

For a long time the populations of the forest were deadened on the fact the forest is a layer of wild fruit, but also a fish pond of many tropical produce edible fruits and nuts with potential as new crop plants, which can be easily domesticated and exploited. A large variety of oilseeds and pulses, including groundnuts, melon and *Irvingia gabonensis *seeds respectively grown well in Cameroun, forming part of the traditional diets of many people.

*Irvingia gabonensis *(Aubry Lecomte ex O'Rorket) is a commercially and indigenous fruit tree of West and Central Africa, which has been identified as the most important tree for domestication. The kernels of *Irvingia gabonensis *are widely marketed domestically, nationally and between these countries [1]. In Cameroon the kernels are used as condiment and are highly valued for their food thickening properties to prepare "ndo'o" or "draw soup" as well as groundnut or njansang (*Ricinodendron heudelotii *) [2]. The chemical properties of the kernels have been the subject of earlier studies [3]. But the majority of studies are base on the ethno-botanic character of *Irvingia gabonensis *tree. Works available shown that, seeds contain protein and fat [4]. Fat is the most abundant component of kernels (70%) particularly two saturated fatty acids: 51.87% of myristic acid (C14:0) and 38.44% of lauric acid (C12:0)[5].

The role of dietary saturated fatty acids on the plasma level and low-density lipoprotein (LDL) metabolism, have been investigated mainly in animals and humans [6,7]. Saturated fatty acids, like myristic acid is generally considered to induce the increase in plasma cholesterol, especially in the LDL-cholesterol concentration [8]. In most study which lead to this conclusion lauric, myristic and palmitic acids such as dairy fat and tropical oils, were considered the most noxious of fats. It is well known that this various fatty acids in the diet exert different affects on serum lipid and lipoprotein concentrations. Saturated fatty acids are thought to increase cardiovascular disease risk because they elevate serum total and LDL-cholesterol concentrations relative to monounsaturated and polyunsaturated [9]. More recently, several studies on a variety of N-myristoylated protein suggest that myristic acid may have different roles when attached to different acceptor proteins [10]. It's most obvious and simple function is contributing to the association of modified cytosolic protein face of membranes. Moreover, Loison [11] reported that, myristic acid seems to be also an important cell component since numerous proteins need to be myristoylated and structural positioning. Dika nut fat has recently become popular in the West and central African sub-region due to their high fat and protein contain. Despite, the fact that kernels are rich in oil they are not exploited yet on a large scale beside the high-output. Conventional sources such as the palm tree oil or leguminous plant seeds. Moreover, the oil of *Irvingia gabonensis *is 90% made up of saturated fatty acid and knowing that saturated fat increases the cholesterol concentration, in particular LDL-cholesterol and expose us to the risks of the cardiovascular diseases [9]. The aim of this study was to test the hypothesis that dika nut fat (5.14 and 13.48%) has no undesirable effects on the plasma cholesterol of rats.

## 2 - Materials and methods

### 2.1. Chemicals

Kits for cholesterol, triglyceride, HDL-Cholesterol and transaminase assays were purchased from Boehringer-Mannheim (Meylan, France) (CHOD-PAP and GPO-PAP methods) and from Teco diagnostics, Anhein (USA). LDL-cholesterol was calculated by methods of Friedewald and Fredrickson. Whereas serum and hepatic proteins was determined used the Biuret method [12].

### 2.2. Oil extraction and fatty acid analysis

Wild mango kernels were bought at Mfoundi market in the city of Yaoundé (Cameroon). Once at the laboratory of nutrition and food security of the University of Dschang, the teguments and the impurities on kernel was removed using hands, then crushed and the flour was macerated in hexane during 48 hours and filtered with Wathman paper. The hexane was eliminated with the rotary evaporator and oil collected and preserved at the refrigerator until use.

The extracted oil was hydrolysed and the fatty acids converted to their fatty acid methyl esters derivatives (FAME) using the BF_3 _(Boron Trifluoride) methanol method [13]. The FAMEs were analyzed on a PERICHROM (Péri 2000 Model) gas chromatograph with a flame ionization detector (FID). The esters were separated on a WCOT fused silica 50 m × 0.25 mm ID coating select FAME.

### 2.3. Experimental design

To prepare rats for the experiment, there were nourished and watered *ad libitum *with diet in table 1 proposed hereafter by Telefo [14], for the nutrition of the rats at the University of Dschang. They were fed a standard diet (which contains 0.1% lipids) for 1 week in order to homogenize their body weight before the start of the experiment.

**Table 1:** Composition of rats diets during adaptation period Telefo, [14]

Ingredients	Quantity of ration/kg
Cornstarch	678
Flour of soya	200
Fish meal	100
Flour of bone	10
Soya oil	1
Cooking salt	10
Vitamin & mineral	1

At 3 weeks of age, they were randomly assigned to one of the following four semi-synthetic groups (table 2). The basic composition of these diets (g/kg) of total weight diet was as follows (table 2). The four diet groups differ only in their natural fat component in order to vary the percentage of dika nut fat (5.1 to 13.48%), lauric acid (0.00 to 48.84%) and myristic acid (0.06 to 58.86%) in the diet (table 3). 4 groups of 10 rats were fed with four different diets for 3 weeks for both study, animals received the appropriate amount of test material in the diet *ad libidum*. Fresh diets were provided every day during the experiment period and tap water was provided in water bottles.

**Table 2:** Composition of study diets

Ingredients (g)	Test diets			
	
	Fat control **(IG**_**0**_**)**	Low-dose **(IG**_**1**_**)**	Mid-dose **(IG**_**2**_**)**	High-dose **(IG**_**3**_**)**
Starch	500	568	523	410
Soya bean oil cake	200	200	200	200
Fish protein	100	100	100	100
Sucrose	20	20	20	20
Flour of bone	20	20	20	20
Cellulose	15	15	15	15
Salt (Nacl)	2	2	2	2
Vitamin mix	3	3	3	3
Choline (ml)	2	2	2	2
Soya oil	80	0	0	0
Dika nut fat	0	50	70	120
**Total (g)**	**940**	**978**	**953**	**890**

**Table 3:** Fatty acid composition of test diets (gram of total weight of fat)

Fatty acids	Test diets			
	
	Fat control **(IG**_**0**_**)**	Low-dose **(IG**_**1**_**)**	Mid-dose **(IG**_**2**_**)**	High-dose **(IG**_**3**_**)**
C10:0		0.77	1.08	1.85
C12:0		20.35	28.49	48.84
C14:0	0.06	24.53	34.34	58.86
C16:0	8.81	2.53	3.54	6.07
C18:0	21.96	1.19	1.67	2.86
C18:1		0.25	0.34	0.59
C18:2 n-6	42.29			
C18:3 n-3	5.62			
C20:0	0.30			
C22:0	0.26			
				
UI	0.71	0.39	0.55	0.94
				
SFA (g)	9.42	49.37	69.11	118.48
PUFA (g)	69.86	0.25	0.34	0.59
				
Total weight fat	80	50	70	120

UI: Unidentified

### 2.4. Animal care

The rats were individually caged in elevated stainless-steel wire-mesh cages except during the acclimation period and had free access to food and water. The temperature was maintained at 25°C. At the end of the experiment, the animals have been let 12 h overnight fast, before being sacrificed with the help of the steams of chloroform, their blood collected by cardiac puncture stored in test tubes. Serum was isolated and preserved at -20°C for clinical pathology parameters. Thereafter, the abdomen of every animal was opened by a midline incision the following tissues were taken liver, heart, spleen, lungs and the kidneys. After been appropriating excised, they were washed in a solution of NaCl (0.9%), wrung and then weighed respectively. With the help of a mortar, 2 g of liver have been ground in 10 ml of NaCl 0.9%. The ground obtain has been centrifuged 10 min at 3000 g and supernatant was recovered in sterile test tubes and preserved in freezer at -20°C. The collected blood was led during 6 hours to the temperature of the laboratory (about 25°C) in a bassinet containing ice, to increase the formation of the blood clot with liberation of the serum. This mixture has been centrifuged 10 min at 3000 g to separate the two phases. Then, remaining phase also call supernatant (serum) have been recovered in sterile test tubes, and preserved at the freezers to (-20°C). These samples have served for the clinical parameter chosen as: total cholesterol, HDL-Cholesterol, triglycerides, the LDL-Cholesterol, serum proteins, hepatic proteins and the transaminase.

### 2.5. Mortality and sign of toxicity

All animals were observed for mortality and gross signs of toxicity twice daily (morning 7.am and afternoon 7 pm). Detailed physical examinations of each animal were made prior to the study and weekly during the study period. Observation included general condition, skin, fur, eyes, nose, oral cavity, abdomen, external genitalia, evaluation of respiration, and palpation for masses.

### 2.6. Body weight and food consumption

Body weight and food consumption were measured over successive periods of 5 days, as the treatment started and every 3 days during the experiment period. Weight and food consumption were measured from rats on days 0, 1, 2, 3, 4, 8, 11, 14, 17, 20, and 23 at the same time until sacrifice. The quantity of food consumed was measured per cage. The intake of test substance per kg body weight was calculated from the nominal dietary concentration.

### 2.7. Statistical analysis

The hypothesis groups were determined by ANOVA analysis, using the statistical software Graphpad Instat 3.5 for windows. Data were analysed between fat control and the three treatment groups and between the three test diets respectively. Dunnett's test was performed to determine mean which were significantly different from fat control and Bonferroni test was used to determine whether there is difference between *Irvingia gabonensis *test diets respectively.

## 3. Results

### 3.1. Dika nut oil fatty acid profile

The fatty acids composition of the *Irvingia gabonensis *oil test material is shown in the table 4. This composition shows that dika nut oil is primarily consists of two saturated fatty acids: lauric acid (40.70%) and myristic acid (49.05%). These two fatty acids, associated to capric (1.54%), palmitic (5.06%) and stearic (2.38%) acids account for 98.86% saturated fatty acids. Oleic acid is the single unsaturated fatty acid identified.

**Table 4:** Fatty acids profile of dika nut fat

Symbols	Fatty acid level (%)
C10:0	1.54
C12:0	40.70
C14:0	49.05
C16:0	5.06
C18:0	2.38
C18:1n9	0.49
	
UI	0.78
	
SFA	98.73
UFA	0.49

UI: Unidentified

### 3.2. Physiological status

No adverse effect of treatment was indicated from physical observations. All the animals were in good health after the 3 weeks experimental diet period. Weight gains during the experimental period were different whatever the diet. Consumption during the experimental period was also different in each group.

### 3.2. Food intake

Data relative to the food intake in the different groups of animals during the 3 weeks of treatment are regrouped in the table 5. It follows from this Data, that there is no significant difference, in the food consumption of the rats during the 3 weeks of experience in comparison to control, the IG_1 _diet in male groups increases significantly (P < 0,01) than fat control groups during the first week of experiment. We also observe that the experimental diets in male groups were consumed slightly more than fat control group, whereas in the female groups it's less consumed. Between the treated animals, we have notice a significantly rise of food consumption (P < 0,05): the first week of experiment in female and male groups rats for the IG_2 _diets respectively; the second week in the male groups rats for the IG_3 _diet and the third week for IG_2 _and in female groups for the IG_3 _diet respectively. The mean food intake between fat control and treated animals were comparable for all diets. Treated groups with high fat contain is above the other diets for male and female groups respectively.

**Table 5:** Food intake data (g) during experimentation period for male and female rats

	Weeks			
		
Diets	**S**_**1**_	**S**_**2**_	**S**_**3**_	M ± SEM
**Male (n = 5)**				
IG_0_	7.39 ± 3.49	26.89 ± 4.52	40.57 ± 7.24	19.88 ± 14.26
IG_1_	10.28 ± 5.48^a^**	24.82 ± 8.69^a^	30.14 ± 9.36^a^	18.81 ± 11.09^a^
IG_2_	7.60 ± 5.01^b^	27.71 ± 6.69^a,b^	42.36 ± 9.80^b^	20.59 ± 15.53^a^
IG_3_	9.19 ± 3.38^a,b^	30.05 ± 6.32^b^	32.79 ± 10.91^a,b^	21.15 ± 13.87^a^
**Female (n = 5)**				
IG_0_	9.91 ± 4.13	26.88 ± 6.42	44.95 ± 10.96	22.01 ± 15.21
IG_1_	7.72 ± 3.26^a^	28.71 ± 5.44^a^	30.14 ± 9.36^a^	22.97 ± 20.02^a^
IG_2_	11.03 ± 7.29^b^	24.91 ± 5.07^a^	42.36 ± 9.80^a,b^	20.87 ± 13.65^a^
IG_3_	10.18 ± 3.60^a,b^	27.43 ± 7.34^a^	32.79 ± 10.91^b^	20.92 ± 12.86^a^

S_1_, S_2 _and S_3 _(week 1, week 2 and week 3 respectively

IG_0_: control diet with 80 g of soya fat; IG_1_: test 1 diet with 50 g of dika nut fat

IG_2_: test2 diet with 70 g of dika nut fat; IG_3_: test3 diet with 120 g of dika nut fat

In the columns, the values affected of the same letter are not significantly different (P > 0.05) and the value with different letter are significantly different (P < 0.05). (Bonferroni between treated animals).

* Significantly different from fat control in the same sex (P < 0.05). (Dunnett's)

**significantly different from fat control in the same sex (p < 0.01). (Dunnett's)

### 3.3. Food consumption

Data relative to indices food consumption are represented in table 6. We noticed from this data, that the indices decrease with age. The added of dika nut fat in the diet of the rats decreases this parameter. The decrease is significantly different (P < 0.05), for IG_3 _diet than control in male groups during the first week of the treatment. During the period treatment, this indices increase with the test diets and it's significantly higher (P < 0.01) for IG_2 _diet than controls in female groups. While, between the test diets this parameter was not significantly different during the whole period of treatment.

**Table 6:** Food indices consumption data (g) during treated period for male and female

Diets	Weeks			
		
	**S**_**1**_	**S**_**2**_	**S**_**3**_	M ± SEM
**Male (n = 5)**				
IG_0_	1.11 ± 0.65	1.00 ± 0.34	0.72 ± 0.10	1.00 ± 0.52
IG_1_	1.32 ± 0.80^a^	0.89 ± 0.23^a^	0.56 ± 0.23^a^	1.04 ± 0.66^a^
IG_2_	1.44 ± 1.15^a^	0.93 ± 0.31^a^	0.69 ± 0.13^a^	1.14 ± 0.88^a^
IG_3_	1.82 ± 1.71^a^*	0.95 ± 0.38^a^	0.61 ± 0.19^a^	1.32 ± 1.33^a^
**Female (n = 5)**				
IG_0_	1.25 ± 0.61	0.83 ± 0.16	0.77 ± 0.22	1.02 ± 0.50
IG_1_	1.58 ± .19^a^	0.94 ± 0.24^a^	0.87 ± 0.27^a^	1.26 ± 0.92^a^
IG_2_	1.83 ± 1.14^a^**	1.04 ± 0.54^a^	0.77 ± 0.17^a^	1.38 ± 0.97^a^**
IG_3_	1.49 ± 1.01^a^	0.87 ± 0.25^a^	0.70 ± 0.13^a^	1.15 ± 0.80^a^

In the columns, the values affected of the same letter are not significantly different (P > 0.05) and the value with different letter are significantly different (P < 0.05). (Bonferroni between treated animals).

* Significantly different from fat control in the same sex (P < 0.05). (Dunnett's)

**significantly different from fat control in the same sex (p < 0.01). (Dunnett's)

### 3.4. Body weights

**Figure 1:**
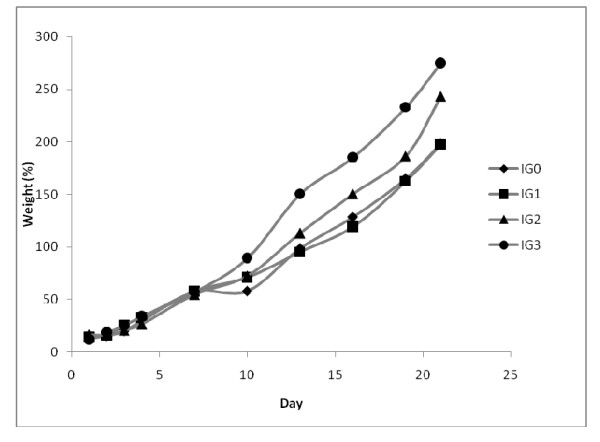
**Male body weight gain (%) during experimental period **. Each point represents a mean between 5 observations

**Figure 2:**
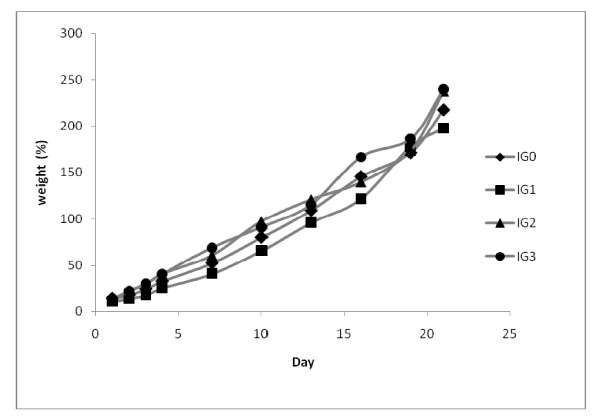
**Female body weight gain (%) during experimental period **. Each point represents a mean between 5 observations

The weight gain (%) is positive and comparable in the IG_0 _and IG_1 _diets of males. However, the body weight gain of rat increases with age for both sexes (Figures 1 and 2). The weight of the rats feeding high dose fat is superior to those of controls. This increase of weight is significantly higher (P < 0.01) than controls for IG_3_; IG_1 _and IG_3 _in male and female diet groups respectively. The weight gain (%) for the IG_2 _diet for female is significantly higher (P < 0.05) than fat controls, while between the test diets this increase of weight is significantly different (P < 0.05) for IG_3 _than IG_1 _diets respectively. The body weight gain of rats is increasing and regular, with high fat treated rats above the other groups.

### 3.5. Organ weights and macroscopic pathology

The results of the table 7, showed a higher significant reduction (P < 0.01) of the weight of the liver in treated male rats compare to fat control. Reversely this reduction was not significantly different for females who consume the same oil. In general, the vital organ weight doesn't vary much from fat control for both sexes, except for the spleen in diet IG_1 _for female that was significantly higher (P < 0.05) than fat control, while between the test diets we don't notice any significant difference from vital organ weight.

**Table 7:** Weight gain (%) during the three weeks treatment for males and females

Diets	Weeks			
		
	**S**_**1**_	**S**_**2**_	**S**_**3**_	M ± SEM
**Male (n = 5)**				
IG_0_	27.12 ± 18.11	95.28 ± 38.75	181.85 ± 49.95	78.52 ± 68.27
IG_1_	29.8 ± 18.01^a^	95.15 ± 35.30^a^	180.08 ± 57.01^a^	79.43 ± 67.19^a^
IG_2_	24.62 ± 19.04^a^	112.15 ± 47.67^a,b^	215.08 ± 61.56^a,b^	90.17 ± 83.77^a^
IG_3_	29.5 ± 20.47^a^	141.65 ± 48.22^b^**	253.96 ± 49.11^b^**	108.03 ± 95.67^a^**
**Female (n = 5)**				
IG_0_	29.22 ± 15.65	26.88 ± 6.42	194.12 ± 31.25	86.85 ± 69.31
IG_1_	21.74 ± 13.52^a^**	28.72 ± 5.44^a^	187.58 ± 17.59^a^	76.68 ± 66.78^a^**
IG_2_	32.85 ± 17.84^a,b^	24.92 ± 5.07^a^	205.26 ± 45.39^a,b^	93.20 ± 72.93^a^*
IG_3_	34.82 ± 19.87^b^**	27.44 ± 7.35^a^	216.68 ± 34.59^b^*	98.00 ± 76.67^a^**

In the columns, the values affected of the same letter are not significantly different (P > 0.05) and the value with different letter are significantly different (P < 0.05). (Bonferroni between treated animals).

* Significantly different from fat control in the same sex (P < 0.05). (Dunnett's)

**significantly different from fat control in the same sex (p < 0.01). (Dunnett's)

Macroscopic observation showed differences in caecal contents and intra-abdominal fat deposition. Although there was considerable individual variation within groups, male and female treated with *Irvingia gabonensis *diet also showed lower mean scores (data not shown) for the degree of fat deposition within the abdominal cavity compared to the fat diets control.

### 3.6. Clinical laboratory studies

The data relative to hepatic, serum proteins respectively and the concentration of ALT and AST according to the dika nut fat managed during the three weeks of treatment are consigned in table 8. These results showed us that the concentration of ALT and AST don't significantly change in male groups. However, the decrease of AST was significantly higher (P < 0.05) in female groups than fat control for all diets and between the test diets there weren't any significantly changes observed for both sexes. In the groups of treated animals the decrease was significantly greater (P < 0.001) for IG_3 _than IG_1_, IG_2 _and for IG_2 _and IG_3 _compare to IG_1 _for males and the females diet respectively. This data also showed, a significantly reduction (P < 0.01 and P < 0.05) of serum proteins level for male compare to fat control for IG_1 _and IG_3 _diets respectively. Although, the reduction of serum proteins was significantly higher (P < 0.01) for female in IG_3 _diet, this decrease was also significantly higher (P < 0.01) for IG_2 _than IG_1 _diet respectively. Whereas, in female treated groups no statistical difference were observe. In the case of hepatic proteins, it concentration was significantly higher (P < 0.01) than fat control for IG_1 _and IG_3 _diets males groups respectively. Whereas, in female groups the decrease was significantly higher (P < 0.01 and P < 0.05) than fat control for IG_2_, IG_3 _and IG_1 _diet respectively.

**Table 8:** Influence of the incorporation of *Irvingia gabonensis *fat on the relative weight of some vital organs for male and female rats

Diets	Organs (g)				
	liver	lungs	kidney	spleen	heart
**Male (n = 5)**					
IG_0_	5.93 ± 0.79	1.00 ± 0.34	1.20 ± 0.26	0.35 ± 0.08	0.57 ± 0.14
IG_1_	4.75 ± 0.50^a^**	0.84 ± 0.05^a^	1.49 ± 0.36^a^	0.36 ± 0.08^a^	0.52 ± 0.03^a^
IG_2_	4.75 ± 0.16^a^**	0.82 ± 0.11^a^	1.09 ± 0.28^a^	0.28 ± 0.04^a^	0.47 ± 0.04^a^
IG_3_	4.85 ± 0.18^a^**	1.28 ± 0.80^a^	1.17 ± 0.30^a^	0.40 ± 0.21^a^	0.54 ± 0.05^a^
**Female (n = 5)**					
IG_0_	0.50 ± 0.22	1.01 ± 0.26	1.33 ± 0.19	0.31 ± 0.07	0.51 ± 0.08
IG_1_	4.98 ± 0.35^a^	1.07 ± 0.23^a^	1.27 ± 0.17^a^	0.42 ± 0.08^a^*	0.54 ± 0.05^a^
IG_2_	4.93 ± 0.32^a^	0.91 ± 0.05^a^	1.34 ± 0.12^a^	0.32 ± 0.06^a^	0.55 ± 0.04^a^
IG_3_	5.07 ± 0.46^a^	0.88 ± 0.13^a^	1.40 ± 0.51^a^	0.32 ± 0.05^a^	0.55 ± 0.07^a^

In the columns, the values affected of the same letter are not significantly different (P > 0.05) and the value with different letter are significantly different (P < 0.05). (Bonferroni between treated animals).

* Significantly different from fat control in the same sex (P < 0.05). (Dunnett's)

**significantly different from fat control in the same sex (p < 0.01). (Dunnett's)

From data in table 9, we can see that the concentration of total cholesterol in IG_2_, IG_3 _and IG_3 _diets increase was significantly higher (P < 0.01) for male and female in comparison to control groups respectively. However, the concentration of triglycerides increases for all test diets compare to control groups. This increase were significantly higher (P < 0.01) in IG_1_, IG_2 _and IG_3 _diets for female rats. Whereas, in male groups this increase was also significantly higher for IG_2 _and IG_3 _than fat control (P < 0.01 and P < 0.05). Concerning LDL-cholesterol concentrations, there were no differences between treated animals in comparison to control and between treated animals, it rise was significantly higher (P < 0.01) for IG_1 _than IG_2 _and IG_3 _diets respectively for male and it decrease was significantly different (P < 0.05) for IG_2 _in comparison to IG_1_; this increase was also significantly higher (P < 0.05) for IG_3 _in comparison to IG_2 _diets for female respectively. These results also show us a comparable rate of LDL-cholesterol concentrations for both sexes. The mean number of total HDL-Cholesterol concentrations showed an increase respectively in the IG_1_, IG_2_, IG_3 _and IG_2_, IG_3 _diets for female and male groups respectively. This increase was significantly higher (P < 0.01) in female treated groups in comparison to control. The concentration of HDL-Cholesterol doesn't also significantly change between treated males, whereas, in female groups this value increase was significantly different (P < 0.01 and P < 0.05) for IG_3 _diet in comparison to IG_1 _and IG_2 _diets respectively. Along the period of treatment we also observed between the animals treated, that the concentrations of total cholesterol was significantly greater (P < 0.001) in IG_2_, IG_3 _diets in comparison to IG_1 _for male and also significantly higher (P < 0.05) for IG_3 _in comparison to IG_2 _diet for female. Statistical mean also showed significantly increase level (P < 0.001) of triglycerides for IG_3 _compare to IG_1 _and IG_2 _for male diets respectively, whereas, in female groups no significantly differences were observed between treated animals. In a general manner, the serum fat contains increase with the rising of *Irvingia gabonensis *fat, but this rising is perfectly observed with animals who receive dika nut fat at high-dose.

**Table 9:** Influence of the incorporation fat *Irvingia gabonensis *on some toxicology parameter for male and female rats

Diets	ALT (U/L)	AST (U/L)	Serum protein (mg/ml)	Hepatic protein (mg/g)
**Male (n = 5)**				
IG_0_	16.36 ± 8.83	26.81 ± 8.02	134.18 ± 18.17	123.04 ± 6.84
IG_1_	17.98 ± 4.36^a^	21.81 ± 0.51^a^	82.03 ± 11.12^a^**	255.70 ± 11.32^a^**
IG_2_	14.44 ± 3.35^a^	17.97 ± 3.35^a^	128.10 ± 21.15^b^	238.99 ± 8.51^a^**
IG_3_	16.29 ± 4.16^a^	33.29 ± 17.24^a^	100.25 ± 9.23^a,b*^	138.23 ± 11.55^b^
**Female (n = 5)**				
IG_0_	10.02 ± 5.89	109.62 ± 43.91	97.72 ± 17.77	333.16 ± 21.08
IG_1_	22.10 ± 10.04^a^	22.68 ± 12.07^a^*	117.47 ± 16.85^a^	293.67 ± 15.50^a^*
IG_2_	7.37 ± 1.02^a^	44.79 ± 34.29^a^*	113.92 ± 10.89^a^	174.69 ± 14.09^a^**
IG_3_	15.32 ± 3.68^a^	38.83 ± 6.27^a^*	141.77 ± 19.53^a^**	206.58 ± 19.49^b^**

AST, aspartate aminotransferase, ALT, alanine aminotransferase

In the columns, the values affected of the same letter are not significantly different (P > 0.05) and the value with different letter are significantly different (P < 0.05). (Bonferroni between treated animals).

* Significantly different from fat control in the same sex (P < 0.05). (Dunnett's)

**significantly different from fat control in the same sex (p < 0.01). (Dunnett's)

## Discussion

Dika nut is a vegetable oil rich in myristic acid. The lauric acid proportion (40.70%) would also classify it among lauric oils which are oils of coconut (45-48%) and palm kernels (54%) [15]. The lauric and myristic acids contents are different from the 58.6% and 33.5% of kernels from Sierra Leone. They are close to 38.44 and 38.80% of lauric acid and 51.87 and 50.60% of myristic acid respectively found in Cameroon sample [5] and Nigeria sample [16].

This composition of dika nut oil justify the fact that this study was conducted to assess the safety of single cell oil rich of saturated fatty acids, when fed by rats during their growing. This way of period treatment in this work is similar to those of Burns [17]. The irregular curve of the food consumption in rats development, justifies itself by the appetizer regulation, that has an effect on adjusting the food hold in order to maintain a balance between the caloric gain and the energy lose for the immediate needs and growth function respectively [18]. This food consumption increases during treated period, despite the augmentation of fat level in the IG_3 _diet. What makes us think that although, at strong level dika nut fat remain appetizing. This increase of appetite could result from an augmentation of the hunger sensation associated with an increase of metabolism. These results came closer to those of Bellis and Magnen [19]. Indeed, the sensory qualities of the food (appearance, flavour, texture) could also have an influence on the intake of food by rats.

The decrease of the food consumption indices during the weeks S_1_, S_2_, and S_3 _of the period of treatment is due to efficient use of foods by rats during their growing. These results are similar to those of Okine and Basarabs [20], on the use of fenugreek on cattle. These same authors also noted that an increase of the food consumption indices decreases the beneficial effects of the food and vice versa.

Body weight gains are near to those obtained by Boozer [21], who found that a diet rich of fat generates an important weight body gain than those with carbohydrates. Likewise, in agreement with the data obtain by Schemmel [22], on rats and mice; a diet rich with fat misleads an elevation of mass adipose which depends on the level of lipids and treatment duration. This effect also depends on the age of the animal, and the species.

The significantly decrease of the weight liver (P < 0.05) for male in test diets, and the significantly increase (P < 0.05) of the hepatic protein rate of these same animals deal respectively with the IG_1 _and IG_2 _diet during 21 days, this also let a doubt on the mechanism action of the fatty acids in dika nut fat. This assertion could be verified with the female rats, despite the absence of modification on the weight liver we also have a decrease of hepatic protein rate in comparison to fat control, while between the animals tested we don't observe any variation on weight of vital organs.

The reduction of the hepatic protein concentrations, recorded with the IG_1_, IG_2 _and IG_3 _diets for female in comparison to control could be bound to a slowing or an inhibition of protein synthesis. That could be attributed: either to the absence of digestive essential amino acid in these diets; either to the not induction of the synthesis enzyme present in dika nut fat that interacted with it activities; either by deterioration initiated by fatty acids that may be in excess in the liver. Brady [23], had gotten similar results on obese rats. On the other hand, the increase of this parameter with the IG_1_, IG_2_, and IG_3 _diets for male in comparison to control, which leads to a gain of weight may, came from a bodily protein synthesis. The high concentration of proteins in IG_1 _and IG_2 _diets for male in comparison to control could be explain by an increase secretion of regulation hormones allowing them to resist poisonous effect of dika nut fat. The significantly decrease (P < 0.01) of protein rate, observed in female groups for IG_2 _and IG_3 _diets could be due to the fact that, at this dose dika nut fat act either by inhibiting protein synthesis, following by the brusque modification of food habit by the administration of *Irvingia gabonensis *fat in diets that was not consumed previously by the animals. The increase rate of serum proteins observed with in IG_1_; IG_2 _and IG_3_, in female groups justify itself: either by the absence of cellular membrane deterioration following the non exhibition of this membrane to the poisonous substances or fatty acids present in the wild mango kernels [24]. On the other hand in male groups the decrease of this parameter in IG_1_, IG_2 _and IG_3 _diets are in correlation to the increase of the hepatic protein concentrations of these same animals. But, the mechanism by which the rate of proteins decrease remains unknown in the limits of this work.

The slight decrease concentrations of ALT in serum for IG_2 _and IG_3 _diets for male groups indicate that the liver has not been clinically affected during the treatment. This assertion is justified by the increase of the hepatic protein concentrations. On the other side, we observed an increase of this parameter in female groups for the treated diet in comparison to control. The significantly decrease (P < 0.05) of AST concentrations in IG_1_, IG_2 _and IG_1_, IG_2_, and IG_3 _for male and female could be respectively due to the non necrosis of hepatic membrane and the absence of modification of the permeability membrane which doesn't cause an elevation of the rate of this enzyme in blood [25,26]. The obvious increase of AST concentrations observed for IG_3 _diet in male groups, could be due to hepatic cell lyses at high-fat contain (120 g) during the whole treatment period (21 days).

It's widely reported that saturated fatty acid can significantly alter plasma cholesterol. Dika nut fat is mostly rich of saturated fatty acid in particular myristic (C14:0 49%) and lauric acid (C12:0 41%) respectively. It's incorporation in the diet of young rats of 28 days showed us that increasing dose (5.10; 7.34 and 13.48%) in treated animals influences in different manner serum lipids. This increase of total cholesterol concentrations in the treated animals in comparison to controls is in relation to the presence in the fat of short chain fatty acids as myristic and lauric acid respectively; also known to generally induce the important increase in plasma cholesterol level specially the LDL-cholesterol concentrations. Nevertheless, a previous study of Loison [11] has shown that when feeding hamster with increasing amount of myristic acid it is interesting to note that the observed modifications in plasma total cholesterol concentrations only reflect variation in the HDL-cholesterol concentration. Even Ngondi [27] study also shown that feeding obese patients with *Irvingia gabonensis *seeds also lead to an increase of HDL-cholesterol. Moreover, in the present study, the significant increase of HDL-cholesterol obtain suggest that in rats, myristic acid although present in high quantities in the diet, is one of saturated fatty acids most responsible for increasing the total plasma concentration. However and contrary to previous studies in human [28,29]. or animals [30,31]. It's fascinating to note that the observed modifications in plasma total cholesterol doesn't only reflect an increase of the total cholesterol concentration, but also an HDL - Cholesterol concentration (table 10) known like being receptor of good cholesterol [32]. This rise is significantly greater (P < 0.01) just in the female group rats for IG3 diets in comparison to control. While between the animals treated increasing amount of *Irvingia gabonensis *fat lead to a significantly increase (P < 0.01 and P < 0.05) on the concentration of HDL-Cholesterol of female rats group for IG3 in comparison to IG1 and IG2 diets respectively. This effect is obviously noteworthy in the light of the known relationship of fatty saturated acid on blood concentration. Salter and Loison [11,33] had both already shown that in hamster increasing amounts of myristic acid in the diet increased the plasma HDL-cholesterol. This is what we observed according to results obtain in this study, that feeding of dika nut fat involves an increase of the HDL - Cholesterol concentration. In this study, it can be suggested that the increase of HDL-C concentration could implicate an inhibition of cholesteryl ester transfer protein activity (CETP) by myristic acid. However, the regulation of CETP activity by fatty acids is probably species-dependent since in man, saturated fatty acids (palmitic acid) increased the activity and the mass of CETP [6]. Consequently, the possible inhibitory effect of myristic acid on the activity or mass of CETP in rats still requires confirmation. Studies on a variety of N-myristoylated protein suggest that myristic acid may have different roles when attached to different acceptor proteins [34]. It's constranslational modification involve in protein-protein interaction as well as in anchoring polypeptides to phospholipid bilayers; The more sophisticated roles for myristic acid include: (a) participation in a "switch" mechanism permitting the protein to cycle in a regulated manner between membranes and cytosol [35-37]; (b) influencing protein conformation, with consequences for protein stability or ligand binding [35]; Thus, the results demonstrated that myristic acid present in *Irvingia gabonensis *oil could be beneficial for the reactions of cellular metabolism, but it action on the increase of HDL - Cholesterol concentration still to be verified. Serum triglyceride metabolism is modulated by changes in the type dietary fatty acids. All the test diets (IG_1_, IG_2 _and IG_3_) containing *Irvingia gabonensis *increased triglyceride concentrations compared to control groups. This increase was significantly higher (P < 0.01) for the IG2 and IG3 diets and significantly greater (P < 0.001) between the treated animals IG3 diets. According to Nicolosi, there exist a linear interrelationship between the increase of myristic acid and the increase of triglycerides concentration. But, Loison also showed that the lauric acid was more efficient in the accumulation of triglycerides. In the case of this study, the increase of triglycerides concentration in male and female rats treated its in correlation to the present of lauric acid (C12:0 40.70%) in dika nut fat. However, the mechanism by which the lauric acid influences the increase of the triglycerides still unknown. In addition and base on previous work of Piot [38], the elongation of lauric acid after its partial oxidization can explain the possible accumulation of the triglycerides in the serum. An eventual inhibitory effect of lauric acid on hepatic triglyceride secretion via the VLDL pathway could also be responsible for TG accumulation [39]. The mechanism by which lauric acid modifies hepatic lipid metabolism is presently unclear. Previous metabolic data have demonstrated that medium chain fatty acids (lauric acid) are preferentially oxidized via the β oxidation pathway and long chain fatty acids are preferentially incorporated into the triglyceride molecule [40].

**Table 10:** Influence of the incorporation of dika nut fat on some clinical parameter for males and females rats

Diets	Total Cholesterol	HDL-cholesterol	Triglycerides	LDL-cholesterol
Mg/dl				
**Male (n = 5)**				
IG_0_	147.56 ± 7.02	82.08 ± 5.68	120.98 ± 9.56	58.232 ± 10.35
IG_1_	134.70 ± 19.65	78.66 ± 6.43^a^	130.24 ± 9.33	43.72 ± 19.58^a^
IG_2_	193.64 ± 7.58^1^**	84.71 ± 2.16^a^	143.58 ± 4.59*	82.57 ± 12.23^b^
IG_3_	186.32 ± 7.85^1^**	85.96 ± 3.53^a^	181.90 ± 14.97^2^**	69.95 ± 9.39^b^
**Female (n = 5)**				
IG_0_	138.10 ± 10.52	65.79 ± 12.67	104.00 ± 16.98	58.71 ± 12.99
IG_1_	160.31 ± 10.18^a,b^	67.10 ± 10.66^a^	137.40 ± 7.61^a^**	65.72 ± 6.39^a^
IG_2_	151.67 ± 6.47^a^	71.86 ± 10.41^a^	157.61 ± 10.65^a^**	48.29 ± 4.63^b^
IG_3_	176.25 ± 21.8^b^**	92.94 ± 5.65^b^**	156.49 ± 11.40^a^**	54.01 ± 6.96^a^

-In the columns, the values affected of the same letter are not significantly different (P > 0.05) and the value with different letter are significantly different (P < 0.05). (Bonferroni between treated animals).

-In the columns, the values affected of the same number are not significantly different (P > 0.05)

^1 ^Significantly higher in comparison to IG_1 _group for male between treated animals (P < 0.001) (Bonferroni)

^2 ^Significantly higher in comparison to IG_1 _and IG_2 _groups for male between treated animals (P < 0.001) (Bonferroni)

* Significantly different from fat control in the same sex (P < 0.05). (Dunnett's)

** Significantly different from fat control in the same sex (p < 0.01). (Dunnett's

## Conclusion

In conclusion, this study shows that increasing amounts of dietary *Irvingia gabonensis *fat (5.10 to 13.48%) in correlation with the rising of myristic acid (23.53 to 58.86%), modify cholesterol metabolism and increase significantly the concentration of HDL-Cholesterol. these data obtained also show that dietary fat lauric acid present in dika nut fat also rising triglyceride accumulation. However, the analysis of the AST and ALT concentration don't show a poisonous effect on behalf of the dika nut fat.

## Competing interests

This work was supported by the "University Agency for Francophonie" (AUF) through its "Network of Researchers in Process Engineering Applied to Agro-Food" (GP3A) by a grant to Hilaire WOMENI.

## Authors' contributions

HMW coordinated the work, as well as prepared the manuscript; FTM was involved in the co-design of the work as well as the draft of the manuscript. NTJ conceived, designed, carried out analytical and statistical analysis. JF and ML also carried out the preparation and the analysis of the fat. All authors read and approved the final manuscript.
